# Does creative thinking contribute to the academic integrity of education students?

**DOI:** 10.3389/fpsyg.2022.925195

**Published:** 2022-08-04

**Authors:** Yovav Eshet, Adva Margaliot

**Affiliations:** ^1^Behavioural Studies Department, Zefat Academic College, Safed, Israel; ^2^Faculty of Sciences, Kibbutzim College of Education, Technology and the Arts, Tel-Aviv, Israel

**Keywords:** creativity, academic integrity, big five-personality, academic dishonesty, creative-thinking preferences

## Abstract

The current research focuses on the nature of the relationship comprising personality traits, creative thinking, and academic integrity. Scholars have confirmed that personality traits and creative thinking correlate positively with academic integrity. However, a discussion of academic integrity, personality traits, and creative thinking is missing in the scholarly literature. This study used a questionnaire survey based on the Big Five Factor to identify personality characteristics, the Academic Integrity Inventory, and the Torrance Tests of Creative Thinking. The sample included 976 students studying in four academic colleges in Israel, of which two are teacher training colleges and two colleges awarding a degree in education. The findings show that most of the students (71%) reported they had cheated at some point during their academic studies, and only one-fifth (21%) thought that they would condemn their peers’ lack of integrity. Creative thinking students and emotionally stable persons tend to be more ethical. Hence, we conclude that it is recommended to deliver creative thinking courses in the teacher’s educational training programs to improve creative thinking levels, reduce academic dishonesty, and create more effective curricula.

## Introduction

The COVID-19 pandemic has affected education, and teacher education, in various ways. As a result of the closure of universities and schools, teachers and students have had to adapt to remote teaching rapidly. Teacher education is no exception. The need to create learning environments for student teachers doing their teacher education preparation implied decisions, choices, and adaptations to meet the expectations of students and the requirements of teacher education (Carrillo and Flores, 2020).

More so, the COVID-19 pandemic has highlighted that creativity is essential for adapting to unexpected events or situations (e.g., other pandemics, conflicts, and earthquakes). For example, creative adaptability has been related to coping behaviors in stressful situations (Orkibi, 2021), or to preparing students to deal with unknown scenarios (Li et al., 2021). Creative teaching for overall learning and development has been established in recent research (Levanon, 2021). Current educational curricula for future educators’ focus mainly on teaching knowledge (content) and developing intellectual skills (Albar and Southcott, 2021). Yet, mere teachers’ content knowledge may not be sufficient (Yenen, 2021). Accordingly, information may be used for the sake of thinking and acting creatively. Studies have revealed that the impact of teachers on the development of young children’s creative thinking skills is considerable (Albar and Southcott, 2021). Hence, creative thinking is considered a primary objective of teaching and learning instruction (Pesout and Nietfeld, 2021). Furthermore, it has also been established as a significant 21st-century skill (Albar and Southcott, 2021) that applies to today’s market and employability (Adegbite and Adeosun, 2021). There is a constantly growing number of professionals, who adapt to new conditions, know how to be creative, and make ethical decisions. These changes challenge the modern notion of education (Niepel et al., 2015). Accordingly, teaching creativity has become an important goal in teacher instruction’s curricula associated with promoting teaching quality (Kimhi and Geronik, 2020).

Creativity is a multifaceted construct articulated differently and traditionally measured as creative thinking (Ivancovsky et al., 2021; Park et al., 2021). Many theories have been developed, focusing on different aspects of it (Sternberg and Karami, 2022). It is generally defined as the ability to produce novel, original responses to task constraints (Ivancovsky et al., 2021). Moreover, creativity has been associated with ethical decision-making (Niepel et al., 2015). It is a prerequisite for cooperating and working, and hence, it may be thought of as a crucial educational goal (Perri et al., 2009 in Niepel et al., 2015). In this context, recent scholarship has stressed the high cost of unethical behavior (e.g., academic dishonesty), including damaging one’s public image, losing someone’s trust, potential legal prosecution, and financial loss (Rengifo and Laham, 2022).

Academic dishonesty is related to the deterioration of educational goals, specifically ideas that impact learners’ intellectual, civic, and psychosocial development. Differently expressed, academic dishonesty prevents students from acquiring and developing integrity and fairness, thus mis-preparing them for their futures (Krou et al., 2021). Recent studies have established that unethical conduct (dishonest behavior) is manifesting on a global scale (Zhang et al., 2021), and more concretely, in the educational sector (Krou et al., 2021). Therefore, the need for qualified teachers who positively impact students’ achievements on the one hand, and utilize proper guided training, on the other hand, is essential (Yenen, 2021).

Research dealing with the interaction between creativity and unethical behavior has admitted inconsistent conclusions (Zhang et al., 2021). Some (Beaussart et al., 2013; Gino and Ariely, 2012; Walczyk et al., 2008; Wang and Si, 2014 in Zhang et al., 2021) have established a link between creativity and dishonesty. Yet, others have found the opposite correlation, arguing that higher creativity is a synonym of ethics, caring, and pragmatic decision-making (Keem et al., 2018; Mumford et al., 2010; Bierly et al., 2009 in Zhang et al., 2021). Furthermore, studies could determine that personality traits play a significant role in creativity. These are mostly contradictory research insights (Jirásek and Sudzina, 2020), which include those concerning the relationship of creativity to ethical misbehavior (Kapoor et al., 2021).

Comprehending future educators’ creativity is crucial (Levanon, 2021) to constructing future pedagogical strategies and preparing teachers for a constantly evolving educational reality (Yenen, 2021; Wu and Kyungsun, 2022). In this context, research on the relationship comprehending creative thinking, personality traits, and academic dishonesty is scanty. Thus, there is a need for a more detailed understanding of the underlying relationship between creative thinking and academic dishonesty. Based on Guilford (1950) Divergent Thinking and Torrance (1966) construct, McCrae and Costa (1987) Big Five Factor construct, the present study investigates the correlation between creative thinking, personality traits, and academic dishonesty in education students. The main research question is: What is the relationship between personality traits, creative thinking, and academic dishonesty among education students?

## Theoretical background

### Creative thinking

Creative thinking is traditionally described as detecting previously unidentified relationships and producing original and novel experiences as a new pattern, including the skill to evaluate, improve, and generate novel solutions (Yang and Zhao, 2021). The research field of creativity measurement is constantly growing (Said-Metwaly et al., 2020). Torrance’s Tests of Creative Thinking is an example of one of the tools scholars in the social sciences that have developed since the 1960s (Said-Metwaly et al., 2017; Park et al., 2021). Based on the notion of divergent thinking, which has been defined as thinking “that goes off in different directions” (Guilford, 1959, p: 381), this is ascribed to the skills that produce multiple ideas connected to remote previous associations, and diverse thinking paths combined to find new alternative and innovative solutions.

Torrance’s Tests of Creative Thinking instrument employs fluency, flexibility, elaboration, and originality dimensions to measure creative thinking behavior (Torrance, 1966). Fluency applies to creating ideas and generating numerous responses. Flexibility applies to cognitive and conceptual diversity and elasticity, namely articulating responses through multiple categories. Elaboration assesses ideas’ level of development and improvement. Originality applies to a unique cognitive skill, and one may also see it as an initial, novel, uncommon, or exceptional, articulating unusual responses. Torrance’s Tests of Creative Thinking has a Verbal and Figural format, each of which has two parallel forms: A and B (Torrance and Haensly, 2003). These tests have been acknowledged in more than 2,000 publications, and recent research is available in more than 35 languages (Said-Metwaly et al., 2020).

Studies in education focus on creativity as a creative potential (Runco et al., 2010; Barbot et al., 2011), namely the skill to produce innovative and valuable solutions. This potential may be thought of as an amalgam of intellectual and personality characteristics, among which one may count divergent thinking abilities, imagination, openness, curiosity, and independence (Anderson and Graham, 2021) and executive functions (Khalil et al., 2019). The relationship comprehending creative capacities (e.g., creative thinking) and personality traits has been the object of considerable research (Theurer et al., 2021). Research has established that creative thinking dispositions are directly determined by personality traits (Ayyildiz and Yilmaz, 2021). For example, Li et al. (2022), found that neuroticism has a negative impact while conscientiousness, openness, and extraversion have a positive impact on creativity. Other research studies have gained contradictory insights (Jirásek and Sudzina, 2020).

### Personality traits

Personality traits represent stable patterns of thoughts, feelings, and behaviors manifest in individuals interacting with their environment (Gouveia et al., 2021). Today, the five-factor model, or FFM, is the most widely used model of personality structure (Sleep et al., 2021). It was developed from a lexical approach using trait descriptive adjectives to identify the structure of personality traits (Gouveia et al., 2021). The FFM categorizes individual personality according to five main dimensions: Openness to experience—reveals the extent of intellectual curiosity, creativity, and inclination for innovation and diversity; conscientiousness—reflects an individual’s propensity to self-discipline, duty, and goal achievement; extraversion—refer to energetic, positively emotional, assertive, friendly, and talkative individuals; agreeableness—refers to likely to show compassion and cooperation toward others rather than suspiciousness and antagonism; and neuroticism (or emotional stability—the person’s propensity to be emotionally stable and to exhibit calm behavior)—reflects the likelihood to experience often unpleasant emotions, such as anger, anxiety, depression, or vulnerability. The FFM is mainly assessed through the Ten-Item Personality Inventory—TIPI (Gosling et al., 2003; Gosling, 2021).

#### Creative thinking and personality traits

As previously stated, creativity (measured as divergent thinking) has been associated with personality traits (Weiss et al., 2021). Thus, multiple studies have addressed the relation of the FFM traits to creativity (Puryear, 2020), but they have not established a systematic nature. Furthermore, recent scholarship has postulated that the above findings may be far from conclusive (Jirásek and Sudzina, 2020). More specifically, it has been argued that the relationship comprising these two is complex and multidimensional (Krumm et al., 2018), leading scholars to contradictory results (Kaspi-Baruch, 2019). However, over the last 20 years, the FFM has become the most dominant approach to assess the nexus of creativity and personality (Puryear, 2020).

It has been argued that *openness to experience* could enhance creative thinking (Kaspi-Baruch, 2019). Scholarly reports show that individuals scoring high on this trait have the skill to produce new, original solutions (Pesout and Nietfeld, 2021). Moreover, previous meta-analytical research has determined that openness to experience (Puryear et al., 2017) is a sound and robust predictor of creativity even when predominantly assessing creativity through idea generation parameters such as divergent thinking tests (Tran et al., 2020). In this context, research concerning the nexus of creativity or divergent thinking with openness to experience (Fürst et al., 2016) establishes positively significant correlations (Theurer et al., 2021). Thus, we posit:

*H1*: The higher the personality trait of openness to experiences, the higher the creative thinking level.

The relationship involving creativity and *conscientiousness* is ambiguous (Kaspi-Baruch, 2019) and has remained equivocal. Some scholars are confident there is a positive relationship, while others contend that the relationship is negative, and finally, others have found no relationship whatsoever (Taggar, 2021). Recent scholarship has shown that separately analyzing creative idea generation (e.g., divergent thinking) versus creative production (e.g., creative achievement) reveals that conscientiousness correlates strongly and positively with creative production measures (Tran et al., 2020). Accordingly, there is a call for a more accurate understanding of the above relationship (Taggar, 2021). Research connecting creativity with low conscientiousness levels has revealed significant positive correlations (Sadana et al., 2021). Thus, we posit:

*H2*: The higher the personality trait of conscientiousness, the higher the creative thinking level.

*Extraversion* is positively correlated with creative thinking (Gocłowska et al., 2019), often entailing higher interactivity and possibility exploration (Tran et al., 2020). In addition, there is scholarly evidence that extraversion represents a strong predictor of creativity (Tohver and Lau, 2020; Giancola et al., 2021). Thus, we posit:

*H3*: The higher the personality trait of extraversion, the higher the creative thinking level.

*Agreeableness* is a negative predictor of creativity (Kaspi-Baruch, 2019). Some studies have attested a negative relationship between this trait and creative accomplishments (Batey et al., 2009). Other scholars showed that high levels of agreeableness are negatively related to creativity (King et al., 1996). However, other studies have found non-significant findings concerning the relationship between agreeableness and creativity (e.g., Furnham and Bachtiar, 2008). Yet, scholars still widely contend that personality is a crucial factor stimulating or hindering creativity (Giancola et al., 2021). Thus, we posit:

*H4*: The higher the personality trait of agreeableness, the lower the creative thinking level.

Although research has not established the relationship between *neuroticism* (reverse-scored as emotional stability) and creativity (Fürst et al., 2016), neuroticism is often considered a negative predictor of creativity (Krumm et al., 2018; Feist, 2019). Some research studies speak of a negative nexus between neuroticism and creativity (Sung and Choi, 2009). However, other research has found non-significant relationships connecting the above (e.g., Berenbaum and Fujita, 1994 in Giancola et al., 2021). As to meta-analysis, a recent study could determine neuroticism’s slight negative effect on creativity (Puryear, 2020). Thus, we posit:

*H5*: The higher the personality trait of emotional stability, the higher the creative thinking.

### Academic dishonesty

Research confirming the ubiquity of academic dishonesty as a normalized student behavior goes back for decades (Krou et al., 2021). The phenomenon of academic dishonesty represents a severe and extensively researched problem in education and psychology. Furthermore, unethical conduct threatens higher education’s integrity (Lee et al., 2020). Personality traits are essential to understanding dishonest behavior (Eshet et al., 2021). In addition, there is a general tendency to believe that creative individuals often tend to engage in and justify unethical conduct (Loesche and Francis, 2020). Despite the extensive literature on the subject (Lee et al., 2020), there is still a gap in the research literature regarding the relationship between the Big Five personality traits and academic dishonesty among education students.

#### Academic dishonesty and personality traits

Studies discussing the predictors of academic dishonesty (e.g., cheating) have argued that personality traits embody general conductive proclivities that impact studying behavior (Lee et al., 2020). In addition, academic dishonesty research has repeatedly employed the personality traits’ model (Peled et al., 2019; Steinberger et al., 2021). It has been revealed that personality determines cheating behavior due to its impact on personal beliefs, and one’s attitude toward learning and studying (Eshet et al., 2021). Studies have shown that personality determines cheating behavior due to its impact on individuals’ beliefs about themselves and others (Steinberger et al., 2021).

Some research has shown that individuals with high levels of openness to experience have negative attitudes toward academic dishonesty (Peled et al., 2019). Other studies have revealed that this trait has an almost insignificant relationship (Giluk and Postlethwaite, 2015; Lee et al., 2020). Furthermore, studies have shown that this trait is a sound predictor of academic integrity (Malesky et al., 2022). Studies on conscientiousness have shown that students who score high on this trait demonstrate a low cheating tendency and can regulate their behavior (Peled et al., 2019; Malesky et al., 2022). Some research has shown that individuals who score high extroversion are more predisposed to cheating behavior (Nguyen and Biderman, 2013). Other studies have revealed that this trait has an almost non-existent relationship with academic dishonesty (Malesky et al., 2022). Agreeableness was found to have controversies on their influences (Giluk and Postlethwaite, 2015; Peled et al., 2019). Studies have found that neuroticism has a relatively null relationship to academic dishonesty (Eshet et al., 2021). Thus, we posit:

*H6*: Personality traits impact academic dishonesty.

### Creative thinking, personality traits, and academic dishonesty

Research indicates that individuals with creative personalities are more likely to engage in unethical behavior, such as cheating (Loesche and Francis, 2020). Nonetheless, the relationship between creative thinking and academic dishonesty has not been extensively examined. The most influential line of research on the relation between creativity and unethical behavior suggests a positive relationship between the constructs (Ścigała et al., 2021). Nevertheless, there is a common perception that people with a high level of creative thinking have a greater propensity for academic integrity. Academic studies have confirmed that students with a high level of creative thinking have a higher tendency to reduce unwanted behavior and improve their academic integrity level (Shane et al., 2018). Based on this, we posit:

*H7*: The higher the creative thinking, the lower the academic dishonesty.

## The research model

**Figure 1:**
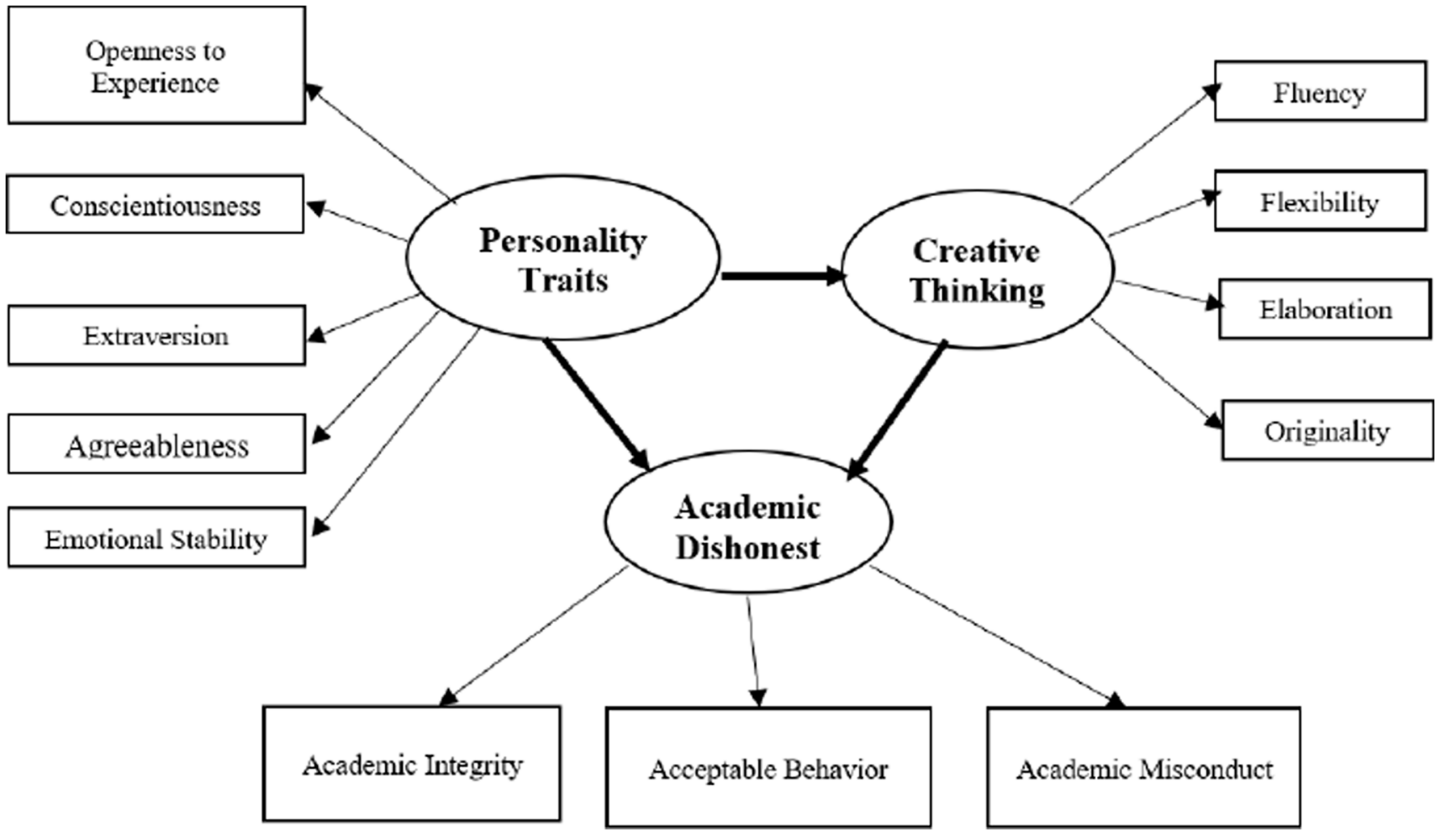
Structural model for creative thinking, personality traits, and academic dishonesty in education students.

As outlined earlier, there is a gap in the research literature regarding the relationship between creative thinking, personality traits, and academic dishonesty among students in general and particularly in education students. Besides, there are not enough studies on the relationship between Torrance’s Tests of Creative Thinking and academic dishonesty, as far as we know. This study sought to elucidate the dimensionality of Torrance’s Tests of Creative Thinking in the Israeli context and its relation to ethical conduct and personality in the academic setting. Thus, based on the literature above, the research model presents the relationship between creative thinking, personality traits, and academic dishonesty (Figure 1).

The research model presents personality traits (measured by extraversion, agreeableness, conscientiousness, openness to experiences, and emotional stability), creative thinking (measured by fluency, flexibility, elaboration, and originality), and academic dishonesty (measured by academic misconduct, academic integrity, and acceptable behavior).

## Methodology

### Participants/sampling

The sample comprised 976 education students from eight academic colleges in Israel. The probability (stratified) random sampling method was employed in data collection. The participants were requested to complete a four-part questionnaire. Eighty-two percent of the students were women and 18% were men. The respondents’ ages ranged from 18 to 44, with a mean of 26.80 years. The participants’ grade point averages ranged from 20 to 100, with a mean of 85.69 points.

### Instruments/materials

#### Creative thinking

Creative thinking was assessed using the Hebrew version of the Torrance Test for Creative Thinking (TTCT) by Runco et al. (2010), which included a drawing question that examines four characteristics: fluency, flexibility, elaboration, and originality. The inter-rater reliability was Kappa = 0.83.

#### Personality traits

The survey employs the Ten Item Personality Inventory (TIPI) scale by Gosling et al. (2003), which comprises 10 items developed to evaluate the personality traits of the participants on a five-point Likert scale, in which 1 means “Not true at all” and 5 “Very true.” Each attribute, in turn, is informed by a double statement. The reliability of this questionnaire as measured by Cronbach’s alpha is questionable (0.82).

#### Academic dishonesty

Academic dishonesty was measured by the Academic Misconduct Scale (Bolin, 2004) and indirectly by the Academic Integrity Inventory (Kisamore et al., 2007). These instruments have been adapted and validated to the Israeli context by Peled et al. (2019). The Academic Misconduct Scale contains 10 items on a five-point Likert scale, in which 1 means “Never” and 5 “Many times.” Its reliability is excellent (0.91 Cronbach’s alpha). The Academic Integrity Inventory consists of 8 items on a five-point Likert scale, in which 1 means “Very unlikely” and 5 “Very likely.” Its reliability is acceptable (0.75 Cronbach’s alpha).

#### Socio-demographic variables

The questionnaire contains a series of socio-demographic items relating to the participants’ age, gender, previous achievements, and course enrolment type.

### Plan of analysis

Complete information maximum likelihood estimates were computed employing the Analysis of Moment Structures (AMOS) program (Arbuckle and Wothke, 1999). The model was examined for the goodness of fit using χ^2^, comparative fit index (CFI), and root mean square error of approximation (RMSEA) fit indices. CFI values above 0.90 and 0.95 indicate adequate and good model fit, respectively, and RMSEA values below 0.08 and 0.05 indicate acceptable and good model fit, respectively (Hu and Bentler, 1999).

## Results

The descriptive statistics and correlations between the research variables are presented in Table 1. A weak negative correlation was found between academic misconduct and the following personality traits: conscientiousness (*r* = −0.229, *p* < 0.01), agreeableness (*r* = −0.160, *p* < 0.01), emotional stability (*r* = −0.145, *p* < 0.01), and openness to experiences (*r* = −0.110, *p* < 0.01). In addition, a weak positive correlation was found between academic integrity and the personality traits: agreeableness (*r* = 0.130, *p* < 0.01), emotional stability (*r* = 0.087, *p* < 0.01), and conscientiousness (*r* = 0.074, *p* < 0.01).

**Table 1:** Descriptive statistics and inter-correlations among variables.

Variables	M	SD	1	2	3	4	5	6	7	8	9	10	11
1. Extraversion	4.69	1.30											
2. Agreeableness	4.87	1.12	−0.023	**–**									
3. Conscientiousness	5.69	1.14	0.132^**^	0.124^**^	**–**								
4. Openness to Experiences	5.12	1.12	0.253^**^	0.099^**^	0.233^**^	**–**							
5. Emotional Stability	4.88	1.27	0.079^*^	0.270^**^	0.304^**^	0.201^**^	**–**						
6. Fluency	10.81	3.70	0.108^**^	−0.019	−0.003	0.100^**^	−0.008	**–**					
7. Flexibility	4.18	1.53	0.048	0.138^**^	0.094^**^	0.125^**^	0.106^**^	0.424^**^	**–**				
8. Elaboration	1.24	0.36	−0.009	0.047	−0.025	0.051	0.004	−0.047	0.157^**^	**–**			
9. Originality	1.15	0.28	0.039	0.044	−0.003	0.081^*^	0.085^**^	0.022	0.305^**^	0.327^**^	**–**		
10. Academic Misconduct	1.44	0.60	−0.045	−0.160^**^	−0.229^**^	−0.110^**^	−0.145^**^	0.063	−0.093^**^	−0.058	−0.068^*^.	**–**	
11. Academic Integrity	3.14	0.57	0.009	0.130^**^	0.074^*^	0.000	0.087^**^	−0.008	−0.033	−0.009	0.014	−0.144^**^	**–**
12. Acceptable Behavior	2.76	0.72	0.005	−0.103^**^	−0.118^**^	−0.028	−0.099^**^	0.015	−0.033	−0.046	−0.075^*^	0.293^**^	−0.069^*^

* *p < *0.05;

** *p< * 0.01;

*n = *976.

A weak negative correlation was found between academic misconduct and creative thinking characteristics: flexibility (*r* = −0.093, *p* < 0.01) and originality (*r* = −0.068, *p* < 0.05). Also, a weak negative correlation was found between acceptable behavior and the creative thinking characteristics originality (*r* = −0.075, *p* < 0.05).

**Figure 2:**
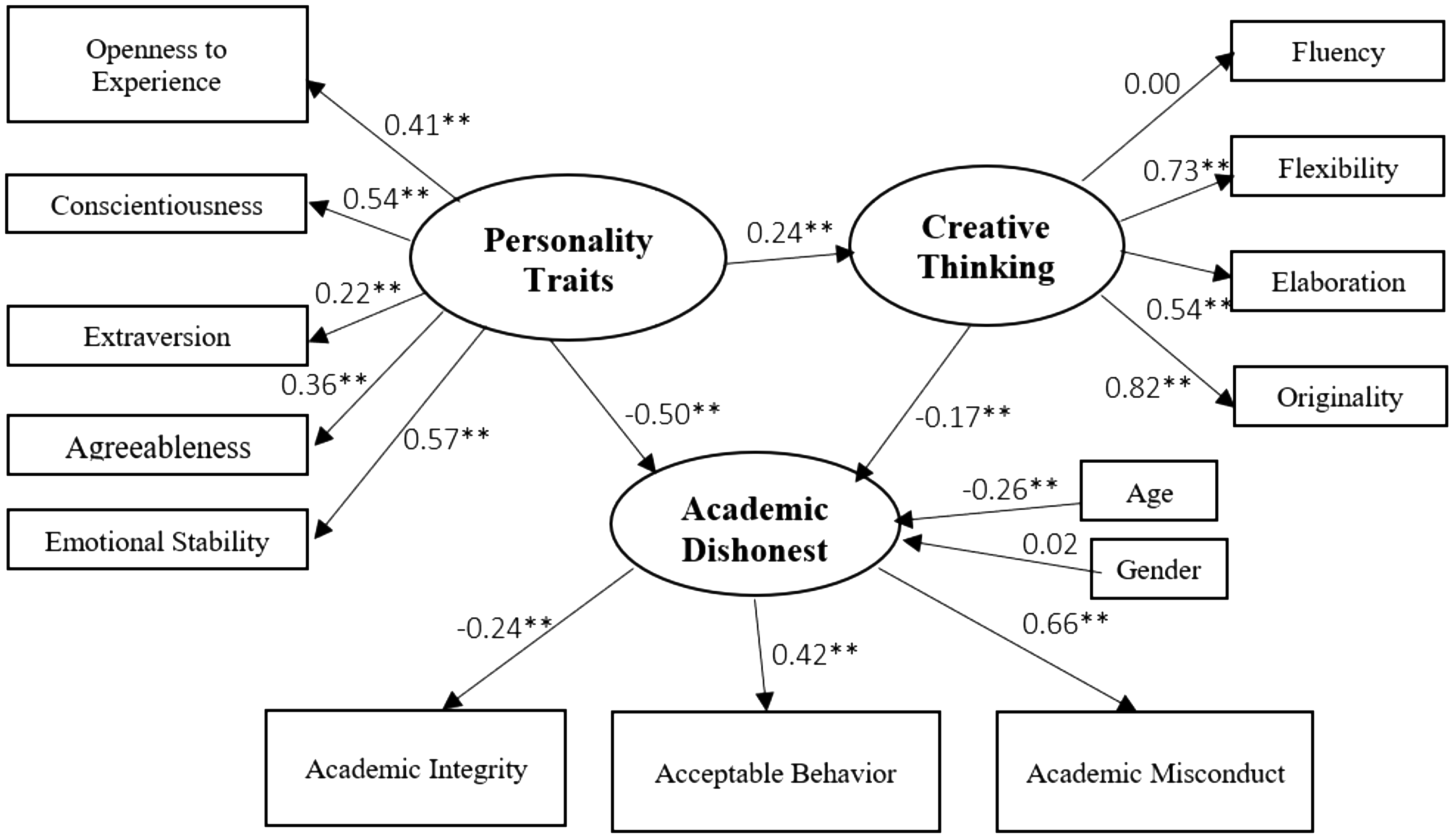
Structural model for determinants of academic dishonesty with standardized coefficients.

Academic dishonesty was modeled by latent variables that measured personality traits and creative thinking. The data fit the academic dishonesty model well (*χ^2^* = 2,120, *N* = 976, *df* = 76, *p* < 0.001, CFI = 0.820, and RMSEA = 0a.081). The R^2^ of the model is 0.32; in other words, the predictors of academic dishonesty explain 32% of the variable’s variance. The structural model is diagrammed in Figure 2.

The analysis results indicate that the variance in academic dishonesty is explained by the research variables related to the Big Five Model: openness to experiences (confirming H_1_), conscientiousness (confirming H_2_), extraversion (confirming H_3_), agreeableness (not confirming H_4_), and emotional stability (confirming H_5_) and to creative thinking: flexibility, elaboration, and originality.

As shown in Figure 2, the traits that impact academic dishonesty are conscientiousness and emotional stability. The total effect of the Big Five personality traits is negative. Among the five personality traits, emotional stability has the most significant impact. The higher the level of emotional stability, the less a person will be engaged in academic misconduct. The rest of the traits have a similar effect on academic dishonesty. The higher the level of each of the following personality traits: extraversion, agreeableness, conscientiousness, and openness to experience, the lower the students’ tendency to cheat. Thus, hypothesis H_6_ is confirmed.

The study also found a negative correlation between age and academic dishonesty, indicating that older students tend to have more academic integrity. Creative thinking was found to have a negative effect on academic dishonesty. In other words, the more creatively a person thinks, the lower will be the level of academic misconduct. Thus, hypothesis H_7_ is confirmed.

## Discussion

The current study examined the relationship between the Big Five personality traits, creative thinking, and academic dishonesty. The study’s results show that this relationship is indeed significant. Furthermore, most of the students (73%) declared that they had engaged in academic dishonesty at some point in their studies. But, only about one-fifth (22%) would condemn this behavior.

In line with the literature (Peled et al., 2019), our study found a significant positive effect of emotional stability on the tendency to engage in academic misconduct. In other words, students with high levels of emotional stability have a lower tendency to academic dishonesty. This may be because students who are high on this trait tend to regulate their emotions and have a sense of security, allowing them to be less influenced by stressful conditions and behave more ethically.

Finally, extraversion was found in this study to have a negative impact on academic dishonesty as well. This result contradicts previous findings (Giluk and Postlethwaite, 2015) that postulated a relationship between the personality trait of extraversion and the tendency to cheat. One possible explanation for this result may be extraversion’s personality trait characteristics, their high motivation to learn, and their proactive personality (John et al., 2020).

The current study also showed a significant impact of the Big Five personality traits on creative thinking. Following previous research (Shi et al., 2016), openness to experience positively affected creative thinking. The personality trait of extraversion was found to have a positive effect on creative thinking as well. This finding is supported by preceding studies (Gocłowska et al., 2019). Interestingly, in contrast to Feist (2019) and Krumm et al. (2018), we found that emotional stability (H_5_) and agreeableness (H_4_) positively affect creative thinking. Hence, the findings from our study refute our hypotheses H_4_ (the higher the personality traits of agreeableness, the lower the creative thinking level) and H_5_ (the higher the personality traits of emotional stability, the lower the creative thinking level). In line with the literature, neuroticism may be positively or negatively related to creativity according to different contexts and environments (Roth et al., 2021). Finally, contributing to the findings of Baer (2017), the personality trait of conscientiousness (H_2_) and creative thinking were found to be positively linked.

Regarding the relationship between creative thinking and academic dishonesty, we found that students with high levels of creative thinking have a higher tendency to academic misconduct. These findings contradict previous scholarly research (Gino and Ariely, 2012; Gutworth and Hunter, 2017), who stated that creative individuals have a higher tendency to academic misconduct. In other words, creative thinking is positively related to academic integrity. These results may be due to the tendency of a creative thinking person to respond to different input creatively and not passively learning together with the self-confidence and ability to succeed at a domain-specific task.

In light of the current study’s findings, we are inclined to agree with Shane et al. (2018) in recommending teaching students to enhance their creative thinking to help them enhance academic integrity. In other words, by enhancing creative-thinking tasks, students will improve their learning skills and avoid unwanted unethical behaviors like academic dishonesty.

## Conclusion and practical implications

As previously stated, creative thinking is among the most sought skills in the 21st century, both at work and in lifelong problem-solving. Teachers and educators oversee preparing the new generation for their future and the demands of their jobs. Although educational institutions are increasingly demanding their professionals utilize Creative Thinking, these same institutions often offer inadequate and underdeveloped training. Furthermore, some scholars stress that the educational system diminishes creativity (Ritter et al., 2020). All the more so, the importance of being creative to adapt to unexpected circumstances (e.g., COVID-19 Pandemics, Crises, Earthquakes, etc.).

Professional knowledge and skills, which are first learned through education training, are vitally important in the teaching profession’s educational context. Teacher training offered at faculties of education plays an essential role in equipping candidates with professional knowledge and skills in all dimensions and supporting their professional development. The process of teacher training directly affects the quality of education youth receive, namely through the quality of teaching (Albar and Southcott, 2021). Therefore, prospective teacher training research is constantly discussed, examined, and re-adapted to new and changing demands. Furthermore, scholarly research based on neural examination suggests that learning mechanisms and creative processes differs across domains (Khalil and Moustafa, 2022). Various standards or competencies are determined to ensure that trained teachers can reach the desired level for the current era (Yenen, 2021).

Additionally, academic integrity is crucial among education students who must act in unique ways to encourage their future students to find their own unique ways of expression and demonstrate their unique skills. In line with the literature, promoting and maintaining academic integrity is a significant concern (Chugh et al., 2021; Eshet et al., 2021). The research literature has not directly investigated the relationship between creative thinking and academic dishonesty. Thus, we sought to examine how education students perceive academic integrity, how they express creative thinking, and how the relationship between the two constructs is expressed. Therefore contributing to the knowledge gaps on concerning the relationship between creative thinking, personality traits, and academic dishonesty. The present study has demonstrated that creative thinking positively impacts academic integrity. Thus, teaching creative thinking reduces academic dishonesty at educational levels and may also reduce any future academic misconduct or professional. In line with the literature, there is a strong positive correlation between Academic Dishonesty and future professional dishonesty (Artiukhov and Liuta, 2017; Eshet et al., 2021).

Our research’s practical implication and main contribution concerns identifying and evaluating the students’ tendency to engage in academic misconduct and their level of creative thinking, which will enable a better understanding of how to support creative thinking and academic integrity in teachers’ training. It is recommended to instruct creative thinking courses in the teacher’s educational training programs to improve creative thinking levels, reduce academic dishonesty, and create more effective curricula.

Following the literature (Yamamoto, 2019), teachers should be willing to use novel methods, ideas, and approaches to stimulate innovation and creativity in their students. For example, teachers can (i) provide opportunities for sparking and enlarging their students’ creative processes, (ii) enhance pedagogies that maximize students’ practices for problem-solving situations where originality and inventive practices develop, (iii) reduce the lecture format and seek natural collaboration and interaction among students/learners, (iv) encourage problem-solving interactions and student autonomy by presenting conflicted learning tasks, and (v) promote problem-solving approaches using real problems and allowing the students to explore new innovative and creative ideas to develop new solutions to real-world problems.

## Limitations and future research

The current study’s limitation is in the sample, which is comprised students who are still in training. The current study did not examine people who have already completed their training and work in actual jobs. In addition, the Academic Integrity Inventory is a self-report questionnaire. Therefore, there is a limited perception of the concept of academic dishonesty among respondents compared to the concept of academic dishonesty defined in the research literature.

Since the current study was conducted with a sample of students in teacher training, it will be interesting and contribute to examining how the relationship between the Big Five personality traits, creative thinking, and academic dishonesty is expressed in teachers’ actual professional performance. Therefore, as a suggestion for future research, we recommend conducting a similar study among teachers who already work in actual pedagogical positions.

## Author’s note

YE is a lecturer at Behavioural Sciences Department, and Digital Learning Designer at Zefat Academic College. His PhD is from Haifa University, Faculty of Management in excellence and outstanding performance. His M.A. is from Haifa University, Public Administration and Policy School. YE’s research areas include Public Administration, Outstanding Employees, Academic Dishonesty. AM, Dean of the Science Faculty in Kibbutzim College of Education, Technology & the Arts; lecturer of Science Education, Creativity, and supervising M.Ed. students in their research projects, developer courses of creative cognition as high order thinking skill, research willingness of students’ academic collaborative writing in online courses and her PhD is from Bar-Ilan University, Faculty of Social Science.

## Data availability statement

The raw data supporting the conclusions of this article will be made available by the authors, upon personal request.

## Ethics statement

Ethical review and approval were not required for the study on human participants in accordance with the local legislation and institutional requirements. Written informed consent from the patients/participants or patients/participants legal guardian/next of kin was not required to participate in this study in accordance with the national legislation and the institutional requirements.

## Author contributions

All authors listed have made a substantial, direct, and intellectual contribution to the work and approved it for publication.

## Conflict of interest

The authors declare that the research was conducted in the absence of any commercial or financial relationships that could be construed as a potential conflict of interest.

## Publisher’s note

All claims expressed in this article are solely those of the authors and do not necessarily represent those of their affiliated organizations, or those of the publisher, the editors and the reviewers. Any product that may be evaluated in this article, or claim that may be made by its manufacturer, is not guaranteed or endorsed by the publisher.
