# Corrigendum: Does creative thinking contribute to the academic integrity of education students?

**DOI:** 10.3389/fpsyg.2023.1274437

**Published:** 2023-10-25

**Authors:** Yovav Eshet, Adva Margaliot

**Affiliations:** ^1^Behavioural Studies Department, Zefat Academic College, Safed, Israel; ^2^Faculty of Sciences, Kibbutzim College of Education, Technology and the Arts, Tel-Aviv, Israel

**Keywords:** creativity, academic integrity, big five-personality, academic dishonesty, creative-thinking preferences

In the published article, there was an error in Figure 1 as published. The “Creative Thinking” ellipse was erroneously switched with the “Personality Traits” ellipse. The corrected Figure 1 appears below.

**Figure 1:**
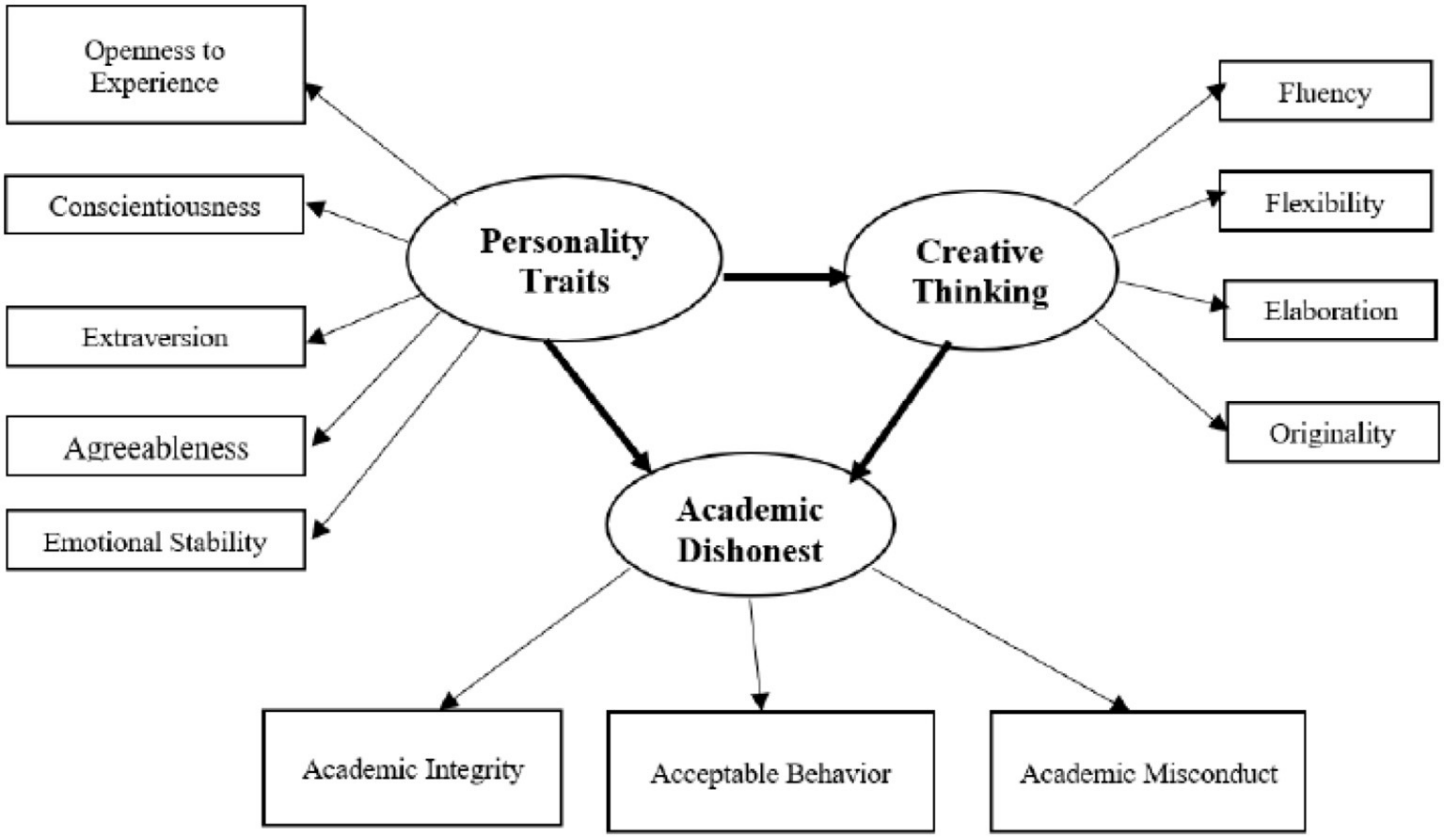
Structural model for creative thinking, personality traits, and academic dishonesty in education students.

The authors apologize for this error and state that this does not change the scientific conclusions of the article in any way. The original article has been updated.

